# KIAA0100 Modulates Cancer Cell Aggression Behavior of MDA-MB-231 through Microtubule and Heat Shock Proteins

**DOI:** 10.3390/cancers10060180

**Published:** 2018-06-04

**Authors:** Zhenyu Zhong, Vaishali Pannu, Matthew Rosenow, Adam Stark, David Spetzler

**Affiliations:** 1 Caris Life Sciences, 4610 S. 44th Pl, Phoenix, AZ 85248, USA; jzhong@carisls.com or zzhong8@asu.edu (Z.Z.); vpannu@carisls.com (V.P.); mrosenow@carisls.com (M.R.); astark@carisls.com (A.S.); 2 Molecular and Cellular Biology, School of Life Sciences, Arizona State University, Tempe, AZ 85287, USA

**Keywords:** KIAA0100, breast cancer, anoikis, microtubule binding protein, heat shock protein, aggression

## Abstract

The *KIAA0100* gene was identified in the human immature myeloid cell line cDNA library. Recent studies have shown that its expression is elevated in breast cancer and associated with more aggressive cancer types as well as poor outcomes. However, its cellular and molecular function is yet to be understood. Here we show that silencing *KIAA0100* by siRNA in the breast cancer cell line MDA-MB-231 significantly reduced the cancer cells’ aggressive behavior, including cell aggregation, reattachment, cell metastasis and invasion. Most importantly, silencing the expression of *KIAA0100* particularly sensitized the quiescent cancer cells in suspension culture to anoikis. Immunoprecipitation, mass spectrometry and immunofluorescence analysis revealed that *KIAA0100* may play multiple roles in the cancer cells, including stabilizing microtubule structure as a microtubule binding protein, and contributing to MDA-MB-231 cells Anoikis resistance by the interaction with stress protein HSPA1A. Our study also implies that the interaction between KIAA0100 and HSPA1A may be targeted for new drug development to specifically induce anoikis cell death in the cancer cell.

## 1. Introduction

Human KIAA0100 protein belongs to the Human Unidentified Gene-Encoded (HUGE) database, which consists of over 2400 novel large human protein genes that have multiple domains potentially capable of binding many kinds of partners [1]. It was identified from a cDNA library in the Kazusa cDNA sequencing project in 1995 [2].

It has been shown that among 71 cancer type-specific tags in previous studies, 28 were specific tags for breast carcinomas [3]. One tag of “GGTCCCCTAC” has been demonstrated highly expressed in the SAGE (Serial Analysis of Gene Expression) libraries of breast carcinomas and breast cancer cell lines compared to the libraries from normal mammary epithelium. The reliable UniGene cluster that matched to the tag of GGTCCCCTAC was Hs.151761, which was found to correspond to the gene *KIAA0100* [4]. In the most recent EST (Expressed Sequence Tag) report, *KIAA0100* was reported to have a total 448 EST sequences from a variety of human tissues, among them the expression of 26 ESTs was confirmed in mammal gland tissue (National Center for Biotechnology Information-NCBI, Unique Gene: *KIAA0100*).

Interestingly, data from multiple studies have shown that *KIAA0100* might be related to breast cancer: in the non-cancerous human mammary breast epithelial cell line MCF10A transformed by v-Src, KIAA0100 protein was significantly up-regulated in response to the malignant transformation by proteomic profiling [5]. The genomic location of *KIAA0100* (17q11) was also found to be within a close proximity to 17q12 chromosomal region. Amplification of this region was found in approximately 25% of breast tumors, which was associated with poor prognosis [6], implying the expression of *KIAA0100* may be affected if such events occur; Also both ERRα and ER-α, were found to be recruited to the promoter region of *KIAA0100* in the mouse model of ERBB2-initiated mammary tumorigenesis [7], implying the expression of KIAA0100 may be potentially up-regulated through these factors in breast cancer. High levels of KIAA0100 expression were also shown to be associated with poor prognosis in patients with invasive ductal breast carcinomas [8]. 

Our recent data-mining from the NCBI Gene Expression Omnibus (GEO) database revealed compelling expression pattern of KIAA0100 in breast cancer patients as well as in *in-vivo* tumor models: the expression level of KIAA0100 was significantly elevated in both basal-like and non-basal like breast cancer compared to normal controls [9,10] (GDS2250), suggesting its involvement in both cancer types. In a mouse HER2 positive breast cancer model, the secondary tumor showed significantly higher expression of KIAA0100 compared to the primary tumor [11] (GDS4099), indicating its expression may be associated to the increasing cancer cells’ aggressive behavior. Meanwhile, multiple bioinformatics tools have been adopted to predict the functions of KIAA0100, and realize that it might be an anti-apoptotic factor related to carcinogenesis or progression [2,12]. Interestingly, our recent study showed that KIAA0100 was elevated in the extracellular vesicles (EVs) fraction in the plasma from breast cancer patients compared to non-cancer controls [13], suggesting KIAA0100 may be linked to EV pathway. However, the molecular and cellular functions that KIAA0100 plays and how it contributes to cancer development, especially in breast cancer cells, remain elusive.

Cancer cell aggression is exhibited in a variety of ways. Cell proliferation/growth is certainly one of those characteristics [14]. However, other aggressive behavior, such as cell anchorage/re-attachment [15], cell adhesion/aggregation [16,17], anoikis resistance [18], a form of apoptosis after the cells’ detachment from the extracellular matrix (ECM), and metastasis/invasion [19], all contribute in demonstrating the aggressive nature of the breast cancer cells. In the current study, we adopted siRNA technology to knock down the expression of KIAA0100 in MDA-MB-231 cells, a highly aggressive triple negative breast cancer cell line [20,21], as a model to study its potential molecular and cellular roles associated with aggressive behavior of breast cancer cells. HEK293 over-expressing KIAA0100 recombinant protein was also employed as an additional model cells to investigate the molecular mechanisms underlying KIAA0100 over-expression and its associated protein interactions.

## 2. Results

### 2.1. Silencing KIAA0100 Expression Does Not Affect the Anchorage-Dependent Cancer Cell Growth/Proliferation

The expressions of KIAA0100 in three different breast cancer cell lines (MCF7, T47D and MDA-MB-231) were first examined by real-time polymerase chain reaction (RT-PCR) and semi-quantitative mass spectrometry analysis (Supplementary Figure S1A–C). All three cell lines were confirmed to express full length KIAA0100 by primers targeted to different regions of the mRNA. Semi-quantitative mass spectrometry analysis showed comparable protein levels of KIAA0100 in these three cell lines. MDA-MB-231 was chosen for the majority of the remaining study because it was widely reported to be the most aggressive breast cancer cell line among the three [20,21].

**Figure 1:**
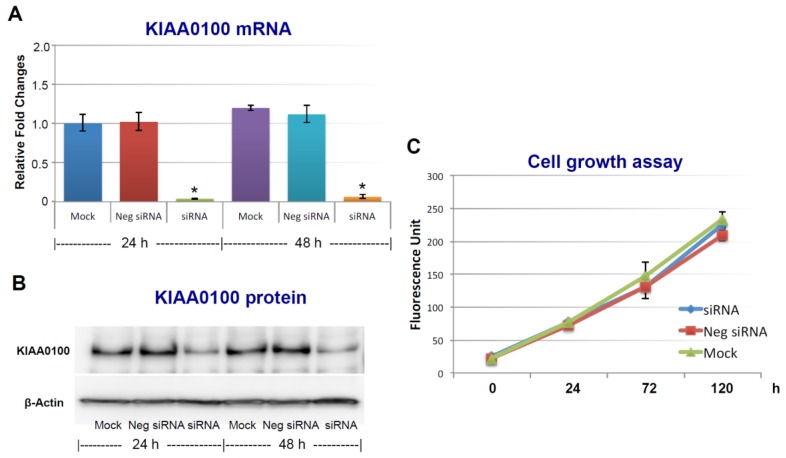
Silencing KIAA0100 expression does not affect cell anchorage-dependent growth/proliferation. MDA-MB-231 cells were first seeded on the culture plate and allowed to settle (attachment); cells were then transfected with KIAA0100 siRNA in a forward-transfection manner. Efficiency of the siRNA was examined by quantitative polymerase chain reaction (qPCR) and Western blot at 24 and 48 h after transfection. (**A**) Relative KIAA0100 mRNA expression was analyzed by ΔΔCt analysis; mock control at 24 h was used as reference group and the β-actin was used as reference gene. Y-axis is relative expression level indicated as fold changes in reference to the mock sample at 24 h. Cells transfected with negative siRNA showed little reduction in KIAA0100 mRNA expression level compared to mock controls (*p* > 0.05); KIAA0100 siRNA treatment significantly reduced the KIAA0100 mRNA expression level by over 90% in the first 24 h and 48 h (*, *p* < 0.05); (**B**) KIAA0100 protein levels detected by Western blot. Consistent with the reduction in the mRNA level, protein level of KIAA0100 significantly decreased within 24 and 48 h, compared to the mock controls and cells treated with negative siRNA; (**C**) cell growth/proliferation were examined by multiTox-Fluor cell viability assay from day 0 to day 5, assay was performed every two days. No significant difference was observed in the cell proliferation/growth of the cells treated with KIAA0100 siRNA and the negative siRNA as well as the mock controls (*p* > 0.05).

To assess the roles of KIAA0100 in breast cancer, its expression in breast cancer cell line MDA-MB-231 was first knocked-down by siRNA targeted to exon 25 in a forward-transfection manner, as indicated in the Methods section below. Anchorage-dependent cell proliferation and growth were then examined, as shown in Figure 1. The expression of KIAA0100 mRNA was effectively reduced more than 90% within 24 h and 48 h after KIAA0100 siRNA treatment compared to mock control and the negative siRNA treated control cells (Figure 1A). Consistently, the protein levels of KIAA0100 were also significantly decreased within 24 h after the treatment, indicating the selected siRNA was very efficient in reducing the expression of both KIAA0100 mRNA and protein levels (Figure 1B). However, there was no significant difference in the anchorage-dependent cell growth/proliferation between KIAA0100-silenced cells and the control cells during the 5-day observation period (Figure 1C), indicating silencing the expression of KIAA0100 had no significant effect on anchorage-dependent cell proliferation/growth in breast cancer cell MDA-MB-231, and also suggesting it might not be directly involved in signal path for the aggressive cancer cell behavior in terms of anchorage-dependent cell proliferation and growth. A similar result was also observed for breast cancer cell line T47D (Supplementary Figure S2A,B).

### 2.2. Scheme of Studying Cancer Cell Aggressive Behavior other than Anchorage-Dependent Proliferation and Growth

**Figure 2:**
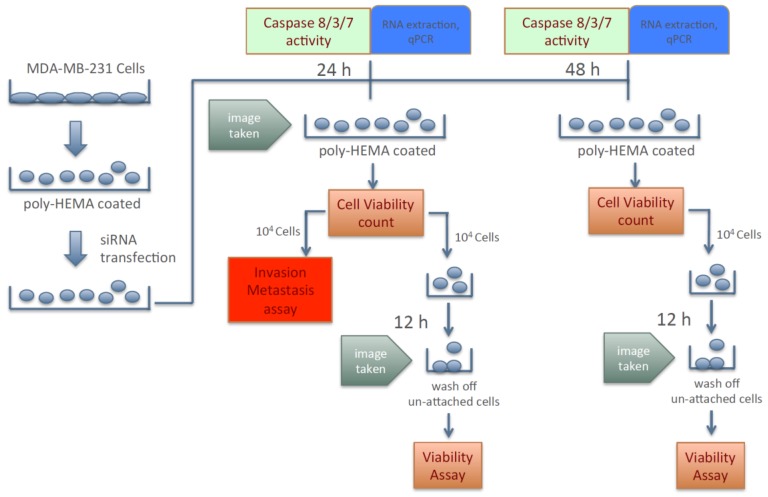
Scheme of assessing breast cancer cells’ aggression behaviors upon silencing expression of KIAA0100.

Because silencing the expression of KIAA0100 failed to show any significant effect in cancer cells in term of anchorage-dependent proliferation/growth, its effect on anchorage-independent behavior of breast cancer cells was investigated. To this effect, cancer cells’ aggregation in suspension, ability to re-attach/re-anchor, anoikis resistance and invasion/metastasis upon KIAA0100 knockdown were first studied in the MDA-MB-231 cells. Figure 2 illustrates the experiment scheme to evaluate these potential effects. MDA-MB-231 cells were maintained in poly-HEMA coated plates, which allowed them to mimic the behavior of cancer cells detached from the ECM and initiating metastasis. Expression of KIAA0100 in the suspended cells was then silenced by the siRNA in a reverse transfection manner. Morphology of the cells (aggregation) in suspension was observed under a light-contrast microscope; total cell number and viability in suspension was then determined by trypan blue based staining on a ViaCell counter (Viability). Cells were then further studied for their ability to attach and migrate as follows: first, cells in suspension culture were seeded to the culture plate without poly-HEMA in 24 and 48 h after siRNA treatment; cells unable to re-attach back to the plate surface were then washed away while cells able to anchor back to the plate were assessed by the viability assay (attachment/anchorage). Second, 24 h after siRNA treatment, the abilities of cells to migrate across the BME (Basement Membrane Extract) barrier was examined (invasion/metastasis). Additionally, cell death in the suspension culture that might be caused by the silencing of KIAA0100 was also evaluated by Caspase 8, 3/7 assay as well as Annexin V staining (anoikis), cells samples were also taken for KIAA0100 protein and mRNA expression analysis.

Therefore, instead of assessing the effects of losing KIAA0100 expression in cells that are well attached, the effect of silencing KIAA0100 on MDA-MB-231 cells in suspension as well as their subsequent behaviors were analyzed independent of the initial anchorage before the KIAA0100 silencing. By doing so, the cells’ aggregation/viability in suspension, anoikis resistance, and ability to re-attach as well as further capability of invasion could then be assessed without interference from the cells’ active growth and they could be evaluated more objectively.

### 2.3. Knocking-Down KIAA0100 Reduced Cancer Cell Aggregation in Suspension

**Figure 3:**
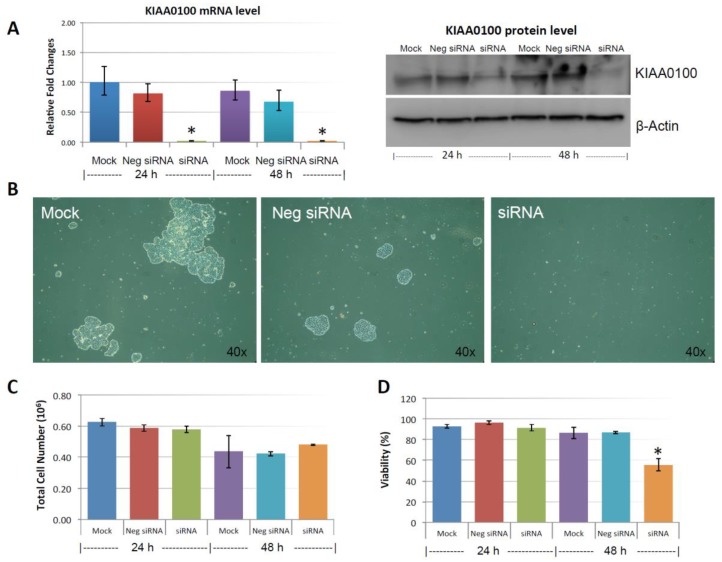
Knocking-down KIAA0100 reduces cell aggregation and viability in suspension. MDA-MB-231 cells were reverse transfected with KIAA0100 siRNA and maintained in suspension culture plate coated with poly-HEMA. (**A**) 24 h and 48 h after transfection, both mRNA and protein levels of KIAA0100 in cell treated with KIAA0100 siRNA were examined by qPCR and Western blot. Both mRNA and protein levels were significantly reduced compared to the cells treated with negative siRNA as well as mock controls (*, *p* < 0.05); (**B**) cell aggregation in suspension upon KIAA0100 silencing was significantly reduced compared to the cells treated with negative siRNA and mock control; (**C**) no significant difference in total cell number counts between cells treated with KIAA0100 siRNA and controls in 24 and 48 h after the treatment by ViaCell (*p* > 0.05); (**D**) cell Viability (in percentage) in 24 and 48 h after treatment. No significant cell viability difference in 24 h (*p* > 0.05). However, silencing the expression of KIAA0100 resulted in significant drop in cell viability 48 h after treatment (*, *p* < 0.05).

Figure 3A shows the silencing efficiency of KIAA0100 by the siRNA in suspended MDA-MB-231 cell culture in a reverse-transfection manner: relative expression levels of KIAA0100 as quantitated by RT-qPCR was reduced more than 90% compared to the mock cell control as well as the cells treated with the negative siRNA; Western blot also showed protein levels of KIAA0100 were dramatically reduced in 24 and 48 h after treatment compared to the mock and the negative siRNA treated control cells, confirming that there was no significant difference in the efficiency of the siRNA treatment between the forward-transfection scheme in the above result and the reverse transfection scheme.

MDA-MB-231 cells were prone to forming loosely connected aggregates when maintained in suspension cultures [22]. Such a cell aggregation mechanism has been shown to play an important role in cancer cell survival during ECM detachment and loss of anchorage [23,24]. Consistent with this notion, control cells including mock and the negative siRNA treated MDA-MB-231 cells formed cell aggregates that were visible under the microscope as shown in Figure 3B. Although the cell aggregates appeared to be different sizes, cells generally were clumped as a small spherical units and the larger aggregates were formed by combination of multiple such units. Interestingly, silencing the expression of KIAA0100 significantly eliminated such cell aggregates within 24 h after the treatment and most of the cancer cells appeared as individual suspended cells. The disappearance of cell aggregation upon KIAA0100 siRNA treatment indicates KIAA0100 may play a role in maintaining cell–cell contact and adhesion under the suspension culture condition.

To make sure such decreases in cell aggregation and viability is not an off-target effect from the siRNA, additional siRNA targeted to KIAA0100 exon 17 was also used as shown in Supplementary Figure S2A,B.

### 2.4. Knocking-Down KIAA0100 Reduced Cancer Cell Viability in Suspension

Unlike other breast cancer cell lines, such as MCF7 and T47D that could form very tight mammospheres in suspension, cell aggregates formed by MDA-MB-231 in suspension culture were not very tight. This suggested that the cell–cell adhesion in these aggregates may not be very strong. It has been shown that by gentle pipetting, cell aggregates formed by MDA-MB-231 in suspension could be separated nicely without any harmful effect on the cells [22]. To take advantage of this, in 24 and 48 h after the KIAA0100 siRNA treatment, cells were then separated by gentle pipetting and the total cell numbers as well as viability were examined through trypan blue staining on a Viacell device. Total cell number regardless of viability showed no significant difference in 24 and 48 h after the siRNA treatment in all samples (*p* > 0.05, Figure 3C). Though a slight decrease in total cell number was observed in all three samples at 48 h, irrespective of the siRNA treatment indicating this effect was not specific to KIAA0100-silenced cells. Therefore, such a reduction was not due to the effect of KIAA0100 knock-down; cell viability percentage was relatively stable at around 90% at 24 h after treatment for all samples. However, at 48 h after treatment, cell viability in KIAA0100 silenced cells was significantly impacted as it decreased to less than 60%. In contrast, no significant difference was observed between the mock and negative siRNA treated control cells, as their cell viability remained relatively the same as that from the respective 24-h time point at about 90%. Total cell numbers were at the similar levels while only KIAA0100-silenced cells showed reduction in cell viability of MDA-MB-231 in suspension, suggesting such a reduction was a direct effect of silencing the expression of KIAA0100 in the suspension cancer cells.

### 2.5. Knocking-Down KIAA0100 Reduced Cancer Cell Invasion/Metastasis

Cell invasion is fundamental to angiogenesis [25], embryonic development [26], immune responses [27], and tumor cell metastasis [28]. MDA-MB-231 cells not only were derived from a metastatic site, but it also has been shown to be a highly invasive breast cancer cell line in multiple studies [29,30,31]. In terms of cancer cell metastasis, the cancer cells must detach from the original site, migrate, re-anchor and invade into distal tissue [18]. As in our design shown in Figure 2, where MDA-MB-231 cells were maintained in suspension, the ability to re-anchor and invade/migrate across ECM could then be assessed by a BME assay, and the effect of silencing KIAA0100 expression on the cancer cells could then be evaluated. To do so, 24 h after the KIAA0100 siRNA treatment, aliquots of cells were collected and re-suspended in serum-free medium. Cell invasion/metastasis potential was examined by their ability to penetrate BME barrier in response to complete medium with 10% FBS.

**Figure 4:**
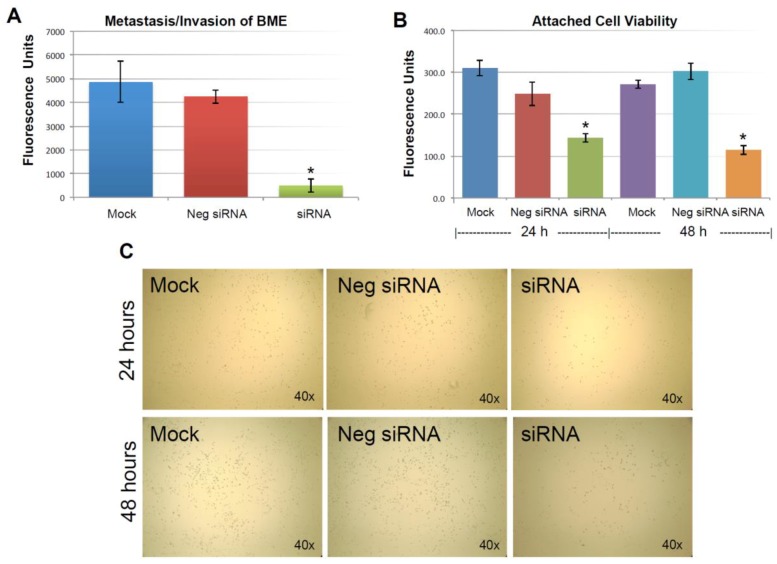
Knocking-down KIAA0100 reduces cell attachment, metastasis and invasion. (**A**) Metastasis/invasion potential were evaluated by the cells’ ability to penetrate BME (Basement Membrane Extract) barrier. Cells able to penetrate the BME barrier were significantly reduced in KIAA0100 knock-down cells, about 90% reduction compared to mock control and 80% compared to cells transfected with negative siRNA (*, *p* < 0.05); (**B**) amount of cells able to attach onto the culture surface were further examined by the cell viability assay. Significant fewer cells were found to re-attach to the surface from KIAA0100 silenced sample compared to control cells (*, *p* < 0.05); (**C**) phase-contrast microscope image, less density of the KIAA0100-silenced cells were observed compared to controls cells in 24 and 48 h after treatment.

As described previously, the expression of KIAA0100 was significantly reduced in 24 h after the siRNA treatment. However, total cell number and viability at this point were not affected. Equal numbers of cells at this time point from each sample were then used for the BME assay. Figure 4A shows MDA-MB-231 cells that were able to penetrate the BME barrier in mock control and negative siRNA transfected cells were at a similar level without significant difference (*p* > 0.05). In contrast, silencing the expression of KIAA0100 significantly reduced the number of cells that were able to migrate across the BME barrier, at times by more than 80%, in comparison to the control cells (*p* < 0.05), indicating that the capability of re-anchoring and invading/migrating across the ECM matrix in the KIAA0100 silenced MDA-MB-231 cancer cells in suspension was significantly reduced. This suggests KIAA0100 might play a role in the epithelial to mesenchymal transition of MDA-MB-231 cells as well as their capability to colonize distant metastatic sites.

### 2.6. Knocking-Down KIAA0100 Reduced Cancer Cell Re-Attachment

The anchorage-dependence of cellular growth and survival prevents inappropriate cell growth or survival in ectopic environments and serves as a potential barrier to metastasis of cancer cells [32]. Cancer aggression is demonstrated by its ability to detach from the original site; on the other hand, cancer cell’s ability to re-attach and start proliferation in the distant site could also be an indicator of cancer aggression. The above BME invasion assay evaluated the cancer cells re-attachment and invasion abilities at the same time. To further investigate the specific effect of silencing KIAA0100 on the cancer cells’ ability to re-attach back to the culture surface, an equal number of MDA-MB-231 cells in suspension culture were seeded to the culture plate without poly-HEMA 24 h later after the siRNA treatment. Cells that were unable to re-attach back to the surface were washed away, while cells that were able to re-attach back to the plate were examined by a cell viability assay.

As shown in Figure 4B, viable cells that were able to re-attach to the culture plate decreased by more than 40% compared to control cells in 24 h after treatment (*p* < 0.05); at 48 h after the treatment, the number of viable cells that were able to re-attach back on the culture plate was reduced by more than 60% compared to control cells (*p* < 0.05). Consistently, light-contrast microscope images show reduced cell density upon KIAA0100 knock-down as compared to the controls under the microscope as shown in Figure 4C, indicating loss of KIAA0100 expression was able to reduce the cells’ ability in re-attaching themselves back to the surface, suggesting that KIAA0100 may play a role in regulating the transition of cancer cells from suspension to anchorage.

### 2.7. Knocking-Down KIAA0100 Sensitized Cancer Cells to Anoikis

Cell invasion and reattachment assays indicated that silencing the expression of KIAA0100 in MDA-MB-231 breast cancer cells in suspension hampered the ability of cells to re-attach back to the culture surface. At the same time, the reduced cell viability in KIAA0100-silenced cells in suspension also implies KIAA0100 expression knock-down may invoke the potential cell death mechanism. Inhibition of anoikis, a cell-detachment induced apoptosis, is one of the primary requirements for cancer cells to become a malignant phenotype which supports tumor invasion and metastasis [18]. The untransformed mammary epithelial cell line MCF-10A underwent anoikis when detached and cultured in suspension condition [32]. In contrast, MDA-MB-231 has been shown significantly higher anoikis resistance compared to other less aggressive breast cancer cell lines upon detachment from the ECM, which is consistent with its aggressive invasion and metastasis capabilities [33].

**Figure 5:**
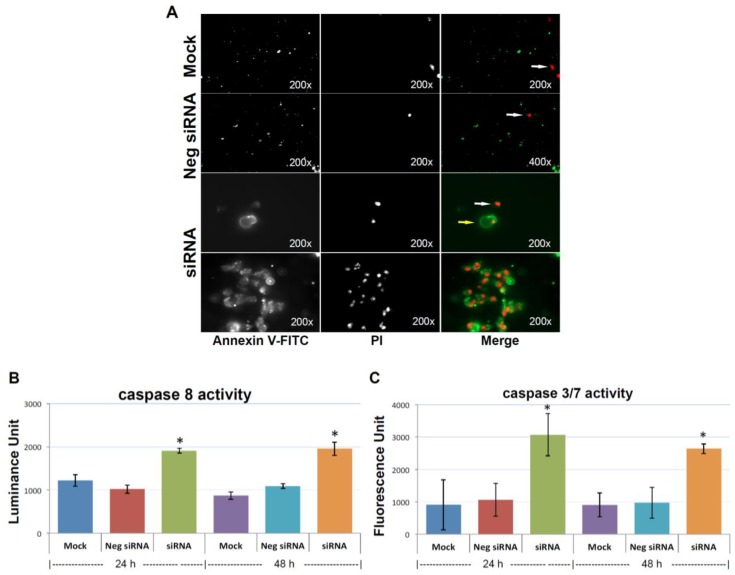
Knocking-down KIAA0100 induces anoikis. (**A**) Annexin V staining: anoikis of the cell in suspension were assessed by Annexin V-FITC apoptosis assay. Most of the mock control and negative siRNA transfected cells show no visible signal of Annexin V-FITC other than sporadic death cell stained with propidium iodide (PI) (white arrow). Two types of staining pattern for Anoikis were shown for cells transfected with KIAA0100 siRNA: early stage of Anoikis—cells that have lost membrane integrity will show red PI staining throughout the nuclei and a ring-like green staining Annexin V-FITC on the plasma membrane; late stage of Anoikis—cells stained with PI for the nuclei without Annexin V staining with or without halo-green like Annexin V-FITC staining. Apoptosis/Anoikis activation was examined by Caspase 8, 3/7 activity assay: (**B**) caspase 8 activity significantly increased in cells treated with KIAA0100 siRNA in both 24 and 48 h after transfection compared to mock control cells and cells treated with negative siRNA (*, *p* < 0.05); (**C**) caspase 3/7 activities in cells treated with KIAA0100 siRNA were significantly higher compared to mock control cells and cells treated with negative siRNA (*, *p* < 0.05).

To find out whether depletion of KIAA0100 could compromise cancer cells, anoikis resistance potential and result in cell death in suspension, anoikis was detected by Annexin V/PI (Propidium Iodide) staining 48 h after the treatment of KIAA0100 siRNA, with the result corresponding to the time-point showing maximal protein depletion. As shown in Figure 5A, MDA-MB-231 cells in suspension showed no Annexin V staining, with only occasional dead cells shown with staining of PI in controls cells (white arrow pointed), indicating negligible anoikis induction in control cells, which is consistent with the previous finding that MDA-MB-231 cells were highly resistant to anoikis [33].

In the initial stage of apoptosis/anoikis, cells relocate phosphatidylserine (PS) from the inner face of the plasma membrane to the cell surface and are detectable by staining with a fluorescent conjugate of Annexin V, and visualized as a ring shape; In the late stage of apoptosis/anoikis, when the cell membrane structure is disrupted, apoptotic cells may appear double positive for Annexin V, as a halo layer and PI staining. Moreover, the late stage of apoptotic cells may be stained rapidly and strongly with PI and may not exhibit Annexin V staining at all [34]. Accordingly, 48 h after cells treated with KIAA0100 siRNA, MDA-MB-231 cells in suspension showed indications of both early and late stage of anoikis, particularly the late-stage anoikis phenotype with halo Annexin V and PI being more dominant (Figure 5A).

To confirm the anoikis pathway was activated, activity for caspase 8 and 3/7 were examined. Caspase 8 was considered activated at the initial stage of Anoikis and at the beginning of the caspase cascade, while caspase 3/7 was more as the effector caspase in later stage [18] in the cascade. Figure 5B shows that both caspase 8 and caspase 3/7 activity were significantly increased compared to control cells (*p* < 0.05) at both 24 and 48 h after the treatment of KIAA0100 siRNA. Therefore, this confirmed that upon the depletion of KIAA0100 in MDA-MB-231 cells in suspension, the anoikis process was initiated. In particular, silencing KIAA0100 induced Anoikis in the suspended MDA-MB-231 cells but not the general apoptosis evidenced by the fact that silencing expression of KIAA0100 on cells that firmly attached to the culture plate did not show similar detrimental effect (Figure 1) as the result here in suspended cells.

### 2.8. Over-Expressed Recombinant KIAA0100 Protein Associates with Proteins from Cytoskeleton and Heat Shock Protein (HSP) Family

Our results have shown that silencing the expression of KIAA0100 in MDA-MB-231 breast cancer cells could have a profound inhibitory effect on the breast cancer cells aggressive behavior, implying KIAA0100 may be involved in supporting the cell–cell adhesion, enhancing anoikis resistance and invasion. However, how it actually accomplishes these diverse roles remains unknown. In order to get deeper insight into the molecular mechanism of how KIAA0100 affects the cancer cells, open reading frame (ORF) of KIAA0100 was cloned with FLAG and Myc peptide tags added to the C-terminus of the recombinant protein. The recombinant plasmid was then stably transfected into cell line HEK293 (as HEK293/pKIA). Over-expressing recombinant KIAA0100 protein in HEK293 cells instead of breast cancer cells may not be ideal, but it is necessary for the investigation due to the technical difficulty in transfecting a large plasmid to multiple breast cancer cell lines, including MDA-MB-231.

**Figure 6:**
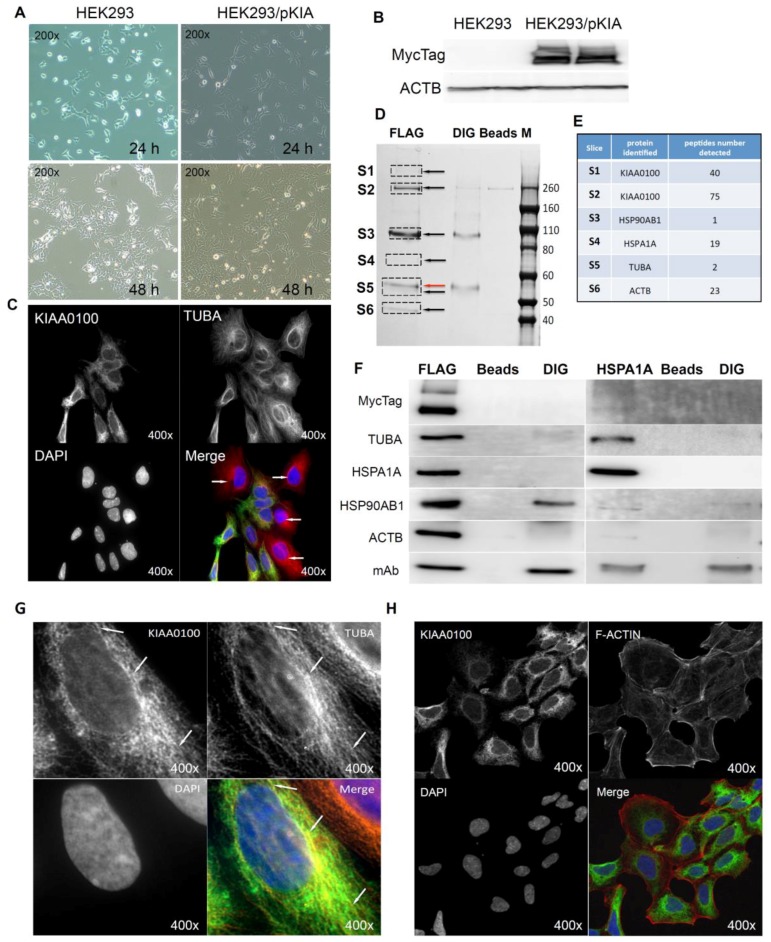

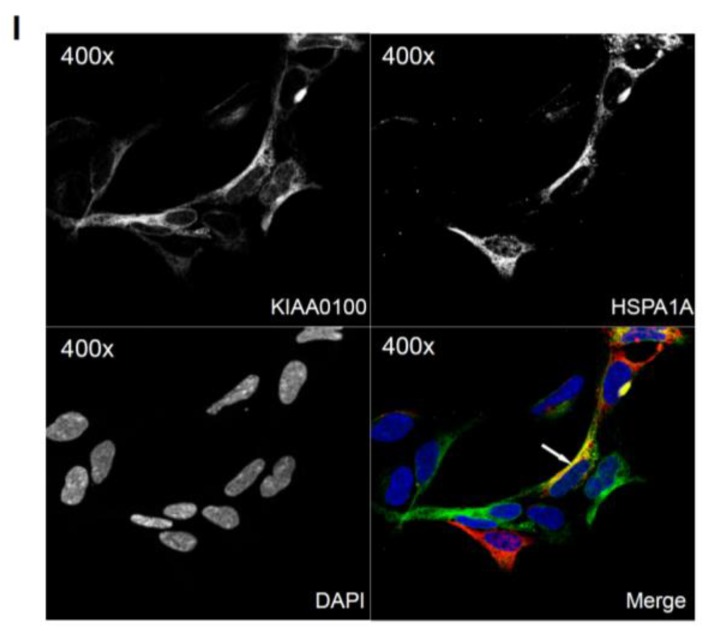
Over-expressing recombinant KIAA0100 protein in HEK293 cells. KIAA0100 over-expressing cell line HEK293/pKIA was established by transfecting KIAA0100 recombinant plasmid to HEK293 cells: (**A**) cell morphology comparison between HEK293 and HEK293/pKIA. 24 and 48 h after seeding same number of cells to the culture plate; (**B**) Western blot confirmed over-expression of the KIAA0100 recombinant protein in HEK293/pKIA cells compared to the parent HEK293 cells; (**C**) immunofluorescence detection of the recombinant KIAA0100 and microtubule network (TUBA) in HEK293/pKIA cells. Cells with white arrow were not expressing recombinant KIAA0100, it served as control for the recombinant KIAA0100 staining; (**D**) recombinant KIAA0100 was captured by anti-FLAG antibody from cell lysate. The captured proteins were separated on 4–12% SDS-PAGE and stained with coomassie blue. Six gel slices were excised and labeled S1 to S6 for mass spectrometry analysis; (**E**) top protein calls from mass spectrometry analysis that match the molecular weight according to the gel slice positions; (**F**) Western blot to confirm the protein identified in the mass spectrometry analysis, IP with anti-FLAG tag antibody and anti-HSPA1A antibody compared to the corresponding controls with anti-DIG antibody as well as empty beads are presented; (**G**) immunofluorescence of recombinant KIAA0100 in HEK293/pKIA cells in a higher magnification, arrow point to the thread-like structure that both appears in the KIAA0100 staining and the TUBA staining; (**H**) immunofluorescence detection recombinant KIAA0100 and F-actin filaments detected by Phalloidin in HEK293/pKIA cells; (**I**) immunofluorescence of HEK293/pKIA cells stained with KIAA0100 and HSPA1A (arrow point to the potential co-localization spot). In all immunofluorescence images above, KIAA0100 showed in green; other co-stained targets showed in red color.

Cells over-expressing KIAA0100 (HEK293/pKIA) were viable with minimal morphological differences in comparison to the original HEK293 (Figure 6A). Expression of recombinant KIAA0100 protein was confirmed by Western blot probed by antibody against the Myc tag added to its C-terminus (Figure 6B). Immunofluorescence with KIAA0100 was performed by mixing HEK293/pKIA cells with the original HEK293 cells (as KIAA0100 staining negative control). As shown in Figure 6C, the over-expressed KIAA0100 recombinant proteins showed a distinguishable localization in the cytoplasm as thread-like networks in HEK293/pKIA cells. Such a localization pattern was highly consistent with the microtubule network stained by the anti-TUBA antibody, implying the recombinant KIAA0100 proteins might be associated with the microtubule network.

Proteins associated with KIAA0100 might provide important information on its potential molecular functions. To find out what proteins might be associated with KIAA0100, protein–protein interactions in HEK293/pKIA cells were first cross-linked by DTBP [35], and the recombinant KIAA0100 protein was then immune-captured by anti-FLAG antibody from the cell lysate. Captured proteins were then separated by sodium dodecyl sulfate polyacrylamide gel electrophoresis (SDS-PAGE) and stained with coomassie blue. Compared to the control IPs, six distinguishable protein bands were found (Figure 6D, S1–S6) with molecular weights of >260 KD, ~260 KD, ~90–100 KD, ~70 KD, ~55 KD and ~45 KD. Protein bands of S1, S4 and S6 are unique to the sample that captured by the anti-FLAG antibody. Protein bands S2 and S3 appeared from IP samples by both anti-FLAG and anti-DIG isotype antibody; however, the intensity of the bands were much stronger in IP by anti-FLAG antibody than the corresponding band from the IP sample captured by the anti-DIG antibody control. Protein band S5 (Figure 6D, red arrow) in the anti-FLAG IP matched the protein band in the control IP sample captured by anti-DIG antibody, whose molecular weight matched the heavy chain of the capture antibody. At the same time, a very faint band (black arrow, S5) underneath that was unique to the anti-FLAG IP was also observed.

**Figure 7:**
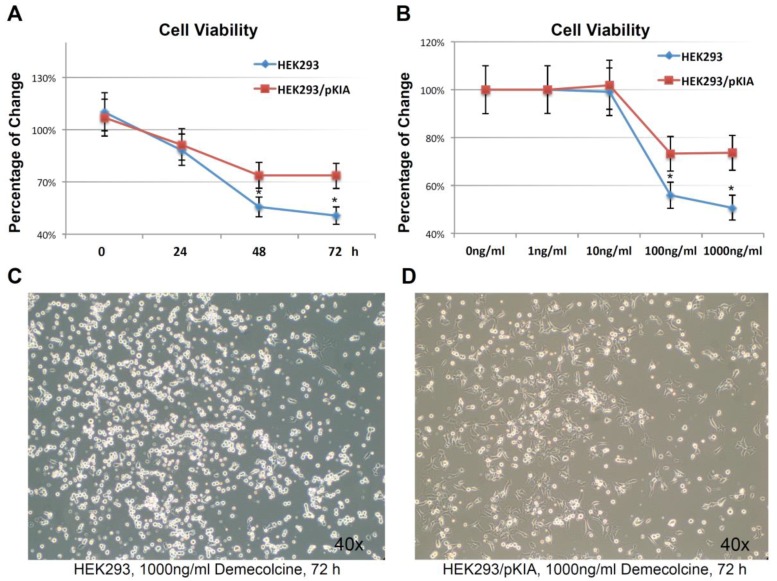
Over-expressing KIAA0100 increases tolerance to microtubule targeted drug. (**A**) HEK293 and HEK293/pKIA cells were seed in 96 well with Demecolcine concentration ranging from 1 ng/mL to 1000 ng/mL, cell viability were examined from 0 (DMSO only) to 72 h. cell viability for HEK293/pKIA at higher dose of Demecolcine (100 and 1000 ng/mL) were significantly higher than the corresponding HEK293 cells (*, *p* < 0.05); (**B**) at 1000 ng/mL Demecolcine treatment, viability of both cells start to decrease at 48 h after treatment, but HEK293/pKIA show significant higher viability compared to HEK293 (*, *p* < 0.05); phase contrast microscope image of (**C**) HEK293 and (**D**) HEK293/pKIA cells treated with 1000 ng/mL Demecolcine at 72 h after treatment. Most HEK293 cells detached from the plate surface and appeared as round shape. In contrast, though some HEK293/pKIA cells also become round shape, most of the cell were still well attached to the plate surface and well spread.

Protein slices of S1 to S6 were then excised from the PAGE gel as shown in Figure 7D according to the area marked by the rectangles respectively, and then subjected to mass spectrometry analysis. The identified protein list from the mass spectrometry was then screened by the expected molecular weight shown on the SDS-PAGE with respective slices. Top proteins that matched respective molecular weight for individual protein slices and number of peptides identified for those proteins are listed in Figure 6E: S1 and S2 were shown to be KIAA0100 as expected with 40 and 75 peptides identified; band S3 was identified to be HSP90AB1 (HSP90) with 1 peptide identified; band S4 was identified to be HSPA1A (HSP70) with 19 peptides; band S5 and S6 was identified to be TUBA with 2 peptides and ACTB with 23 peptides respectively. Therefore, the identified proteins belong to two major families, cytoskeleton proteins and heat shock proteins (HSP).

To verify the finding from the mass spectrometry analysis, the Western blot test was then performed by the antibodies against the identified targets respectively. As shown in Figure 6F (left), KIAA0100 protein was clearly detected by the anti-Myc antibody from the IP sample captured by the anti-FLAG antibody at the positions of S1 and S2, while no recombinant KIAA0100 was detected from IP captured by the antibody isotype and bare beads controls, confirming the IP captured with anti-FLAG antibody was very specific to the recombinant KIAA0100 protein; KIAA0100 recombinant protein was detected at around ~260 KD, consistent to its calculated molecular weight. The signal of the recombinant protein was also detected at the S1 position (>260 KD), which might be a result of larger protein complex formed by KIAA0100 with other proteins, or due to potential post-translational modification. The cytoskeleton proteins TUBA and ACTB were detected at the corresponding position of band S5 (black arrow) and S6 in sample captured by anti-FLAG IP while none of them was detected in the control IPs; the protein corresponding to gel slice S4 was confirmed to be HSPA1A by the Western blot. TUBA, ACTB and HSPA1A were confirmed without any non-specific capture from control IPs, suggesting that these proteins were most likely co-precipitated with the recombinant KIAA0100 proteins. In contrast, the protein corresponding to slice S3 at ~90 KD was confirmed to be HSP90AB1 by the Western blot, but moderate non-specific capture was also observed by IP with isotype antibody. This is because the IP input-capture monoclonal antibodies were at a similar level, suggesting the much higher HSP90AB1 signal detected in comparison to the control may be partially due to the protein co-precipitated with KIAA0100.

The potential interaction of KIAA0100 with HSPA1A was particularly notable due to its broad involvement in important cellular processes. Interestingly, HSPA1A was also detected in the slice S1, which may be a protein complex formed with KIAA0100, by the mass spectrometry, further indicated its potential interaction with KIAA0100. To confirm its potential association with KIAA0100, IP and Western blots were also performed with anti-HSPA1A antibody as the capture antibody and detected with same set of antibodies as shown in the recombinant KIAA0100 IP. As shown in Figure 6F (right), recombinant KIAA0100 and TUBA proteins were found co-precipitated with HSPA1A without non-specific capture in control IPs. In contrast, the co-precipitated HSP90AB1 signal was found not so different from the isotype control IP, and therefore it is most likely HSP90AB1 was not involved in the interaction among KIAA0100, TUBA and HSPA1A. Although ACTB was also co-precipitated by HSPA1A antibody, the signal was relatively low compared to that from recombinant KIAA0100 IP, suggesting the interaction that mainly consists of KIAA0100, HSPA1A and TUBA, may be independent from the relatively minor association of KIAA0100 to HSP90AB1 and ACTB.

The initial KIAA0100 immunofluorescence in HEK293/pKIA cells in Figure 6C shows the localization pattern of that KIAA0100 similar to the microtubule network; immunoprecipitation, mass spectrometry and Western blot then confirmed the association between KIAA0100 and the microtubule protein TUBA. To further substantiate this observation, the individual HEK293/pKIA cells co-stained with KIAA0100 and TUBA were enlarged in Figure 6G. KIAA0100 proteins showed the thread-like network structures. Some but not all of the recombinant proteins were aligned to the same structures stained with the anti-TUBA antibody, which is consistent with the IP and earlier immunofluorescence results. At the same time, co-immunofluorescence was also performed with anti-KIAA0100 and Phalloidin, a dye that specifically recognized the cell F-actin structure. As shown in Figure 6H, F-actin structures shown by Phalloidin staining were mainly shown as fibers on the edge of the cell body with minimal pattern similarity and overlapping with the pattern of KIAA0100.

Because the IP, mass spectrometry and Western blot results strongly indicated the interaction between the recombinant KIAA0100 protein and the stress protein HSPA1A, their cellular localization was also determined by IF (Immunofluorescence), where HEK293/pKIA cells were co-labeled with KIAA0100 and HSPA1A antibodies. Consistent with the IP result, Figure 6I shows that KIAA0100 also partially co-localized with HSPA1A, which strongly suggests the cellular existence of such interaction in the KIAA0100 over-expressing model cells.

Over-expressed recombinant KIAA0100 proteins showed microtubule-like network structures and its interaction with TUBA, one of the explanations could be that the KIAA0100 protein may act as a microtubules binding protein MBP. If so, over-expressing recombinant KIAA0100 protein in HEK293/pKIA cells might be able to prevent microtubule drugs, such as Demecolcine from accessing the microtubule structure, in turn rendering the cells more tolerant to the microtubule-binding drugs.

To examine this hypothesis, HEK293/pKIA and the parent HEK293 cells were treated with the microtubule depolymerizing drug Demecolcine [36] ranging from 1 ng/mL to 1000 ng/mL concentration, and endpoint cell viabilities were compared at 72 h (Figure 7A). Both cells responded to the Demecolcine treatment in a dose-dependent manner, but the cytotoxicity of Demecolcine on HEK293/pKIA cells were significantly lower than the corresponding parent HEK293 cells: at 100 ng/mL of Demecolcine, the cell viability for HEK293 was at around 56% while viability for HEK293/pKIA above 70% (*p* < 0.05); at 1000 ng/mL of Demecolcine, the cell viability for HEK293 was around 50% while the viability for HEK293/pKIA still stayed at above 70% (*p* < 0.05). Cytotoxicity for both cell lines in response to treatment was also shown in a time-dependent manner: with concentration of 1000 ng/mL (Figure 7B), cells over-expressing KIAA0100 had a slower declining curve over 72 h period compared to the cells without over-expressing the protein. Cytotoxicity was also observed under the light contrast microscope as shown in Figure 7C,D. For the HEK293 cells, 72 h after Demecolcine treatment, most of the cells became round shaped and lost cell anchorage from the surface while most of the HEK293/pKIA cells still remained on the culture plate surface with cell body well spread, consistent to the viability assay above. The results indicate over-expressing KIAA0100 was able to slow down the Demecolcine-induced cytotoxicity, suggesting to some extent the recombinant KIAA0100 proteins did prevent the microtubule depolymerizing drug from accessing the microtubule structure, further implying the recombinant KIAA0100 proteins might be in close contact with the microtubule network.

### 2.9. KIAA0100 Co-Localized with Microtubule Network and HSPA1A in MDA-MB-231

**Figure 8:**
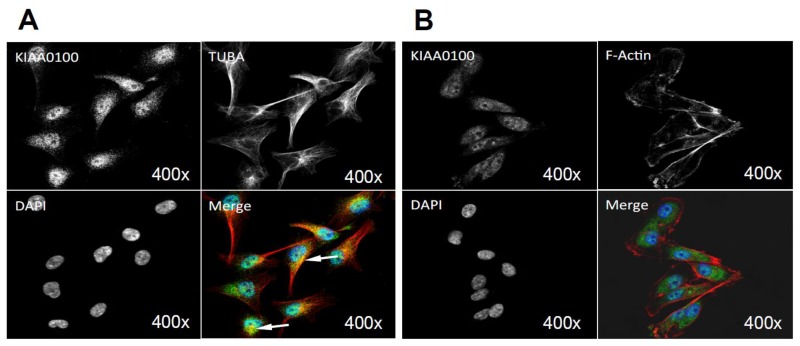

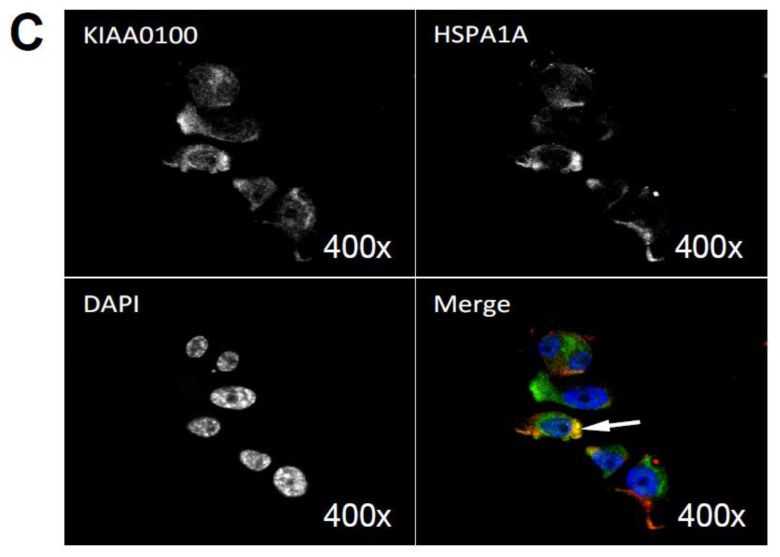
Immunofluorescence of KIAA0100 in MDA-MB-231 cells by con-focal microscope. (**A**) MDA-MB-231 cells stained with of KIAA0100 and microtubules TUBA (arrow point to yellow); (**B**) MDA-MB-231 cells stained with KIAA0100 and Phalloidin (F-actin); (**C**) MDA-MB-231 cells stained with KIAA0100 and HSPA1A (arrow point to the yellow spot). KIAA0100 showed as green, other targets showed in red color.

By using the KIAA0100 over-expressing HEK293 model cells, we were able to identify recombinant KIAA0100 proteins mainly associated with microtubule networks and HSPA1A. In order to verify whether the same interactions could also exist in the breast cancer cell line MDA-MB-231, co-immunofluorescences on MDA-MB-231 cells were performed and observed under the con-focal microscope. As shown in Figure 8A, unlike the recombinant KIAA0100 in HEK293/pKIA cells, the native KIAA0100 proteins in MDA-MB-231 cells showed speckles-like staining. However, the shape of the distribution is roughly consistent with the tubulin staining and partially co-localized (yellow); in contrast, neither staining pattern similarity or co-localization with F-actin staining could be observed (Figure 8B), consistent with the result from KIAA0100 over-expressing HEK293 cells. The co-localization of the KIAA0100 with HSPA1A in MDA-MB-231 cells was also confirmed in Figure 8C, consistent with the above results from KIAA0100 over-expressing HEK293 cells. Our result confirmed that KIAA0100 proteins are mainly associated with microtubule networks and HSPA1A proteins in the breast cancer cell line MDA-MB-231, suggesting such association may reflect its potential functions.

### 2.10. Knocking-Down HSPA1A Sensitizes MDA-MB-231 in Suspension to Anoikis

We have shown a strong association of KIAA0100 protein to microtubule networks and HSPA1A in KIAA0100 over-expressed HEK293 and MDA-MB-231 cells. Because microtubule networks and HSPA1A were both reported to be involved in anoikis resistance, we then wondered which protein might be more responsible for the anoikis effect that we observed in the suspended MDA-MB-231 cells upon KIAA0100 silencing. Therefore, MDA-MB-231 cells in suspension were treated with either siRNA to silence the expression of HSPA1A, or Demecolcine to de-polymerize the microtubule networks. The respective effects were then observed and compared to the KIAA0100 silenced cells in above results.

**Figure 9:**
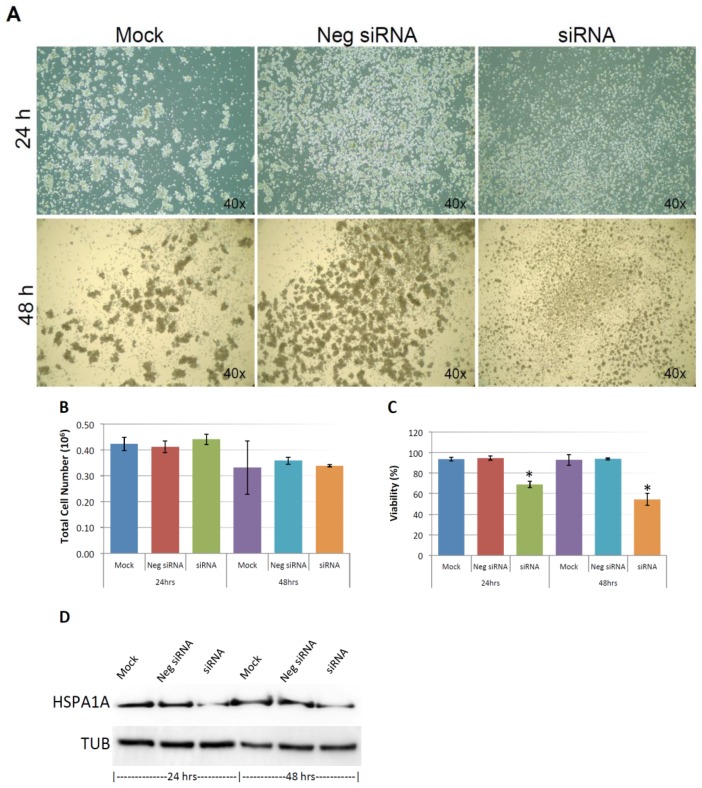
Knocking-down HSPA1A showed similar effect compared to silencing the expression of KIAA0100 in MDA-MB-231 cells. (**A**) MDA-MB-231 cells were transfected with HSPA1A siRNA, cell morphologies were observed under microscopy at 24 and 48 h after the treatment. Mock and negative siRNA treated cells show significant aggregation in suspension culture in 24 and 48 h, while HSPA1A siRNA treated cells lost cell aggregation from 24 h after the treatment; (**B**) total cell number in the suspension culture were similar at 24 and 48 h between mock, negative siRNA treated cells and the HSPA1A siRNA treated cells; (**C**) viability of the HSPA1A treated cell dropped to about 70% in 24 h and then below 60% in 48 h; (**D**) Silencing the expression of HSPA1A by the siRNA was confirmed by Western blot analysis.

**Figure 10:**
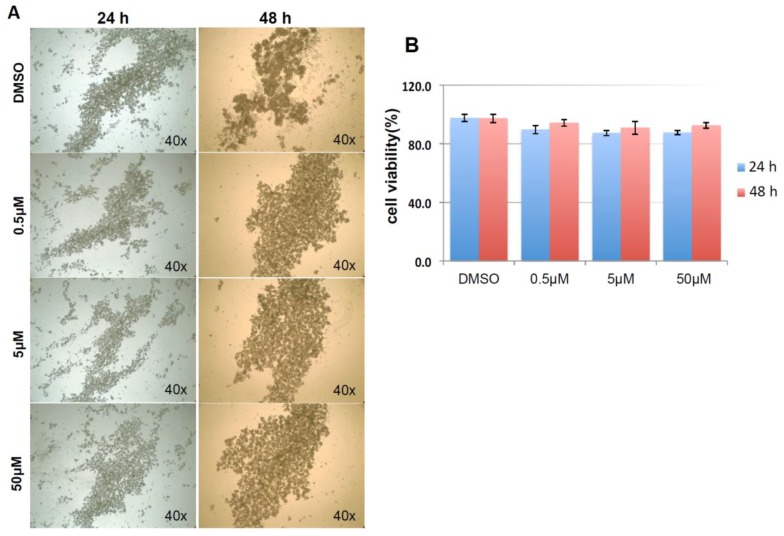
Demecolcine treatments on MDA-MB-231 in suspension culture. (**A**) Morphologies for MDA-MB-231 treated with 0.5, 5, 50 µM of Demecolcine for 24 and 48 h. There was not much difference in morphologies in terms of cell aggregation between control DMSO treated cells and the cell treated with different concentration of Demecolcine; (**B**) cell viability, both cells treated with DMSO and the cells treated with different concentration of Demecolcine show high cells viability in 24 and 48 h after the treatment with no significant difference.

As shown in Figure 9A, and similar to the above result, mock and negative control siRNA-treated control cells formed visible cell aggregates in suspension, but the aggregation dramatically dropped in cells treated with HSPA1A siRNA within 24 h after the treatment, the loss of cell aggregation became more obvious 48 h after treatment. This phenomenon was very similar to the result above that diminishing cell aggregation in KIAA0100 silenced cells. Consistent with the morphology changes, cell viabilities of HSPA1A-silenced cells also dropped dramatically to about 70% in 24 h and below 60% in 48 h after treatment, which was correlated to the drop in the expression of HSPA1A. No significant change in total cell number compared to controls was observed (Figure 9B,C,D). Such a drop in cell viability was also observed in the cells with KIAA0100 siRNA treatment, suggesting KIAA0100 and HSPA1A might be involved in the same pathway of anoikis resistance in suspended MDA-MB-231 cells. In contrast, treating cells with varying concentration of Demecolcine (0.5, 5, 50 µM) did not show a similar effect of losing cell aggregation and viability (Figure 10A,B) on the cells, indicating depolymerization of microtubules alone in the suspended MDA-MB-231 cells was not enough to introduce anoikis, suggesting that the association of KIAA0100 with HSPA1A but not microtubule might be more likely responsible for the anoikis resistance in the breast cancer cell line MDA-MB-231. Silencing KIAA0100, which resulted in a loss of interaction partner to HSPA1A, may be the cause of activating the anoikis pathway, further implying that the interaction between KIAA0100 and HSPA1A may account for the anoikis resistance for MDA-MB-231 cells.

## 3. Discussion

Breast tumorigenesis is a multi-step process starting from benign and atypical hyperplasia progressing into in situ carcinoma, invasive carcinomas, and culminating in metastatic disease [37]. The development and progression of breast cancer are involved in a complex process including interaction of many genetic, epigenetic and environmental factors at different stages of cancer cells, such as proliferation/growth, anchorage/re-attachment, adhesion/aggregation, anoikis resistance, and metastasis/invasion. KIAA0100 has been found to be elevated in breast cancer tissues from multiple studies [4,8,9,10] as well as in malignant transformed benign breast cells [5]; in addition, our previous study also showed that KIAA0100 protein was found in all three different breast cancer cell lines including MCF7, T47D and MDA-MB-231 as well as their respective extracellular vesicles (EVs) preparations [13], suggesting this protein might be related to breast cancer aggression.

In the current study, the role of KIAA0100 in cancer aggression was investigated by adopting as a model MDA-MB-231 cells—a highly aggressive breast cancer cell line [20,21]. We found that while depleting the expression of KIAA0100 did not affect cell anchorage-dependent proliferation/growth, it did show a significant detrimental effect on the aggressive behavior in anchorage-independent cells. In suspension, the aggressive behavior of breast cancer cells such as cell aggregation, re-attachment, migration and invasion was affected upon silencing the expression of KIAA0100 to different degrees, suggesting KIAA0100 could be involved in a broad regulation network. In particular, we found that silencing the expression of KIAA0100 renders the cancer cells in suspension sensitive to anoikis. We also provided evidence that KIAA0100 may be involved in anoikis resistance through its interaction with HSPA1A. At the same time KIAA0100 might also regulate other cancer aggression behavior through its interaction with microtubule networks.

Silencing the expression of KIAA0100 reduced multiple cancer cell aggressive behaviors in MDA-MB-231. However, understanding which behavior was triggered first, or whether they were all activated at the same time remains unknown, partially due to the fact that these seemingly separate processes are actually intertwined and they may have mutual influence, which made it more difficult to isolate individual events experimentally. For instance, it has been shown that cell aggregation was important for anoikis resistance and promotes survival [23,24], which in turn would affect the cells’ capability of regaining anchorage. On the other hand, if cells were under anoikis, it would be reasonable to assume that the cell–cell adhesion and their re-anchoring ability would be also interrupted. However, because MDA-MB-231 cells were reported to be anoikis-resistant [33] and its cell aggregates in suspension are relatively loose compared to other breast cancer cell lines [22]; therefore in this case, this may suggest that the cell–cell adhesion/aggregation dependent survival may not be the key contributor for its anoikis resistance, and the initiation of the anoikis process but not the cell aggregation upon the silencing KIAA0100 might be the main driver for the loss of cell viability. Therefore, the loss of cell aggregation might be a consequence of an Anoikis initiation.

We found it interesting that silencing the expression of KIAA0100 in the suspended MDA-MB-231 cells specifically induce anoikis, a programmed cell death process invoking cell detachment from the extracellular matrix unlike canonical apoptosis in well-anchored cells [18]. MDA-MB-231 cells that are firmly attached on the culture plate did not response to the KIAA0100 siRNA treatment for up to 5 days, while the same treatment on the cells in suspension showed response within 24–48 h after the treatment. One potential explanation is that KIAA0100 may be particularly important to promote cell survival under stress conditions caused by losing cell anchorage and it may contribute to the acquisition of anoikis resistance, an important step during cancer cell malignant transformation [18]. In contrast, when a cell is not under stress, such a function may not be that significant or may even be redundant. Our results also showed that the interaction between KIAA0100 and HSPA1A, a stress protein that is well known to be involved in promoting cell survival under stress conditions, is consistent with such interpretation [38,39,40] as well as the outcome from our experiments involving silencing the expression of HSPA1A in the suspended MDA-MB-231 cells.

One limitation of using the KIAA0100 over-expressing HEK293 cells is that the distribution of KIAA0100 may be cell line-specific in MDA-MB-231 or other breast cancer cell lines and appears to be different from HEK293. Native KIAA0100 in breast cancer cell MDA-MB-231 was localized in cytoplasm as speckles (Figure 8A). As shown in Supplementary Figures S3 and S4, native KIAA0100 in other breast cancer cell lines T47D and HEK293 were essentially the same. KIAA0100 has also been detected in multiple non-breast cancer cell lines, such as U-251MG (glioblastoma), A431 (epidermoid carcinoma) and U2-OS (osteosarcoma) cell lines from the Human Protein Atlas database [41] and showed very similar patterns. Therefore, the natural localization of the KIAA0100 protein was not expected to be significantly different across different cell lines. Also, our results in MDA-MB-231 cells suggested that KIAA0100 most likely is involved in the regulation of universal existing pathways such as anoikis and the microtubule network. Therefore, over-expressing KIA0100 in HEK293 to obtain additional substantiation of our experimental result from breast cancer cells and explore them further is not an unusual approach. For example, in the neuroscience field, K562 cells (a leukemia cell line) stably transfected with APP are often used in studying Aβ production and processing and using yeast two-hybrid screenings to implicate biological meaning for human protein-protein interactions [42,43]. In the current study, using KIAA0100 stably-transfected HEK293 cells as an over-expressing cell model is necessary because the transfection of the KIAA0100 plasmid to multiple breast cancer cell lines is very inefficient if it is not impossible due to the large size of the plasmid [44,45] (~11 Kbp) and low transfection efficiency of those cells by different transfection reagents required. With the assumption that a general cellular mechanism of KIAA0100 may be involved, HEK293, which demonstrated a much higher transfection efficiency compared to breast cancer cell lines, became the only solution in our case. Moreover, the identified KIAA0100 interaction with the microtubule network and HSPA1A was in turn confirmed to be co-localized in the breast cancer cell line MDA-MB-231, suggesting the potential molecular mechanism of KIAA0100 in the breast cancer cell line MDA-MB-231 was consistent with the discovery from the HEK293 model cells in our study.

Although speckles, KIAA0100 was noticeably confirmatory to the pattern of the microtubules network structure but not the actin network. In contrast, the over-expressed recombinant KIAA0100 protein formed a clear thread-like network structure and showed partial co-localization with the microtubule network, implying the speckles-like distribution of native KIAA0100 protein might be just protein spots that were distributed sporadically along the microtubule network. This was further supported by the immunoprecipitation that showed tubulin protein, the building block of the microtubule network, was co-immunoprecipitated with the recombinant KIAA0100 protein. Additionally, over-expressing KIAA0100 significantly increased the cells’ resistance to the microtubule depolymerizing drug Demecolcine, which further confirmed the possibility of close contact between KIAA0100 proteins and the microtubule structures.

Microtubules have been proven critical for anoikis resistance, aggregation, migration and invasion [15,16,17,36,46,47]. Our results suggested KIAA0100 might be functioning as a MBP (Microtubule Binding Protein), which have been shown to be a group of structurally and functionally diverse regulators of microtubule-mediated cellular processes [48]. MBPs was reported to either stabilize/destabilize microtubules or move/transport various cargoes along the microtubule networks [48]. MBPs have also been demonstrated to regulate the binding of microtubule-targeting drugs to microtubules; thereby modulating cancer cell sensitivity to these drugs [48,49,50]. Our results showed potentially close contact between KIAA0100 with microtubules was consistent with such a description. On the other hand, whether KIAA0100 is just bound to microtubules subunits or move along the network with the cargoes is still left to be investigated.

Interaction with microtubule network implies KIAA0100 may also have a role in regulating cell spreading, mobility, metastasis and invasion [17,36,51], which are the primary functions of microtubule network. According to the trypan blue cell viability assay, viabilities of suspended MDA-MB-231 cells were similar between controls cells and the KIAA0100 silenced cells at 24 h after treatment; a re-attachment assay by seeding cells at this moment showed that about 50% of the cells were able to re-attach to the culture plate, which was roughly consistent with the cell viability at 48 h after treatment due to the ~12 h time gap needed for the re-attachment assay. This suggests that at least part of the reason for the cells’ inability to re-attach to the culture surface was due to the loss of cell viability, possibly through activating the anoikis process. On another hand, the same cells were also seeded to evaluate their capability of invasion/metastasis potential. One interesting comparison is that between the cell re-attachment rate and the migration/invasion rate upon depletion of KIAA0100: theoretically if sensitizing cells to anoikis was the only effect from silencing the expression of KIAA0100, we might expect a roughly similar percentage of signal reduction in the BME metastasis-invasion assay. Unexpectedly, the BME invasion result showed more than 80% reduction in signal from the KIAA0100-depleted cells compared to controls, indicating that besides its role in anoikis, KIAA0100 may have other functions related to the capability of regaining anchorage as well as migration/invasion and implies this might be accomplished by its interaction with the microtubule network. 

Multiple members in the HSP protein family potentially interacted with KIAA0100, in particular HSPA1A interaction was confirmed as it co-precipitated and co-localized with KIAA0100 in both KIAA0100 over-expressed HEK293 and MDA-MB-231 cells. The HSP protein family has long been shown to be involved in protein-chaperoning functions such as protein folding and refolding [52,53]. On the other hand, HSP proteins have been also widely reported to have stress-induced anti-apoptotic properties [38] and are actively involved in various processes such as tumor cell proliferation, invasion, metastases and death [53]. Interestingly, it has been shown that HSPA1A is required for cancer cell survival under stress conditions but not for cell proliferation and growth [39]. Due to such dual functionality, the question becomes whether the interaction between KIAA0100 and HSP proteins found in the current study was a result of a regular protein folding process through HSP’s chaperone function, or the possibility that KIAA0100 actually modulates anoikis through HSP proteins. From our results, if such interaction is just because of the need for proper protein folding and KIAA0100 was not in the same survival pathway with HSPA1A, it might not be expected that silencing the expression of KIAA0100 could result in such strong effect in sensitizing cancer cells to anoikis, a mechanism known to be inhibited by HSP proteins. Consistently, silencing the expression of HSPA1A showed very similar effect on the MDA-MB-231 cells in suspension compared to the silencing expression of KIAA0100, in terms of losing cell aggregation as well as anoikis resistance, implying KIAA0100 and HSPA1A could be involved in the same signal pathway.

KIAA0100 is a large protein at about 250 KD in size. Naturally large proteins may contain multiple domains and serve as docking platform for other molecules [1,2]. Therefore, it is not impossible that KIAA0100 is able to interact with multiple proteins that belong to a wide array of functional categories. Our immunoprecipitation experiments showed that KIAA0100 interacts with microtubules and HSPs; however, whether they interact all at the same time as a complex or independently remains unknown. Potentially there are four interaction models that could be suggested from the IP results: (1) HSP and KIAA0100 are bound on same microtubule filament but in separated location; (2) HSP, KIAA0100 and microtubule bind to each other as a complex by different domains; (3) HSP interacts with KIAA0100, and KIAA0100 then bind to microtubules; (4) HSP binds to KIAA0100 and KIAA0100 binds to microtubules independently. There are two major domains on HSPA1A protein: the N-terminal nucleotide binding domain (NBD) is responsible for binding and hydrolyzing ATP; the C-terminal substrate-binding domain (SBD) binds to the client/substrate proteins [54]. Interaction network of HSPA1A has also been explored extensively by both experimental and bioinformatics approaches (CORUM: P0DMV8; IntAct: HSPA1A; STRING: HSPA1A) [55,56,57], but so far no study has yet demonstrated a direct interactions between HSPA1A and microtubules. Meanwhile, according to the data from the Human Protein Atlas, HSPA1A proteins are mainly localized to vesicles in the cytoplasm but not distributed along the microtubule networks (Human Protein Atlas: HSPA1A). In addition, silencing HSPA1A led to sensitization of the cells to Anoikis, which is similar to that of silencing KIAA0100 while Demecolcine treatment failed to do so, suggesting the interactions between KIAA0100 with microtubule and HSPs may be independent events. Whether HSPA1A is a cargo carried by KIAA0100 or vice versa still needs to be further investigated.

It has also been shown that HSPA1A was capable of binding to different phospholipids [58] or even embedding itself within the lipid bilayer [59]. One of the phospholipids that has shown high affinity binding with HSPA1A is bis-(monoacylglycerol)-phosphate (BMP) [60]. Concentration of BMP in the late endosome is 15 mol % of the total lipid content of the organelle and can comprise as much as 70 mol % of the lipid composition of the intra-endosomal vesicles [61,62], which is closely related to multi-vesicular bodies (MVB) and EVs generation [63,64]. Indeed, HSPA1A was one of the most common proteins that are found in EVs [65,66,67,68]. Therefore, it raised the possibility that KIAA0100 might be also mediating the intra-vesicle transportation of HSPA1A proteins. MVBs or even EVs might be one of its potential destinations. This is consistent with our previous finding that showed KIAA0100 was detected in EVs of all three breast cancer cell line EVs as well as in a plasma EV fraction from breast cancer patients [13].

Lastly, one interesting implication is that most of the cancer chemotherapy drugs are targeted to cancer cells with active cell cycles because most enzymes that making DNA and RNA would be stalled upon treatment. Because non-cycling cells are allowed to take the time for repairing the damage, DNA-damaging agents will primarily hit cells in the S-phase in mitosis. Hence, cells in the G0/G1 phase of the cell cycle have long been known to be relatively resistant to classical cytotoxic therapy [69]. MDA-MB-231 controls cells in suspension culture were not in an active proliferation status as indicated by negligible increase in total cell number from 24 to 48 h after treatment and the numbers were very close to the initial seeding cell number of 5 × 10^5^ (Figure 3C and Figure 9B). Treatment with Demecolcine did not show significant toxicity to the cells partially because the Demecolcine targets mitosis and may not have a significant effect on quiescent cells. In contrast, invoking of anoikis upon silencing KIAA0100 expression alone suggested that KIAA0100 might be an attractive target aimed at the quiescent (G0/G1) cancer cells. Although HSPA1A-related small molecules drugs have been developed [70] to target similar signaling path, such drugs may be toxic not only to cancer cells, but also to normal cells due to their inhibitory effects on normal cellular function like protein folding, autophagy, and lysosomal function. Because lack of toxicity on anchored cells by silencing KIAA0100 and the association between KIAA0100 and HSPA1A might be responsible for the anoikis resistance in suspended cells, specifically targeting to KIAA0100 or its interaction with HSPA1A might be able to sensitize the cancer cells that are in the process of metastasis to anoikis with less cytotoxicity effect to normal cells.

Unfortunately, most of the basic biochemical characterizations for KIAA0100 are not yet available, such as structure, domains, post-translational process/modification, protein–protein interactions as well as potential activity if there is any. Indeed, this protein is so unique in the human protein database that no other protein has been found to have significant homology to it (NCBI BLAST); and no significant conserved domain could be found from mammalian protein database. Although bioinformatics analysis suggests that KIAA0100 might contain domains like FMP27 and Apt1 in bioinformatics analysis: UniqProt: Q14667, analyzed by InterPro (https://www.ebi.ac.uk/interpro), Pfam (https://pfam.xfam.org/) and SMART (http://smart.embl-heidelberg.de), none of these domains originated from mammalian species. In contrast, those domains were rather from yeast and plant database, which might limit their functional interpretation in mammalian cells. Our finding suggested that there might be a microtubule or HSP protein-binding domains on KIAA0100, which may need to be further explored.

In summary, we investigated the cellular and molecular functions of KIAA0100, a gene that is strongly related to breast cancer aggression in the breast cancer cell line MDA-MB-231. We showed that KIAA0100 might modulate aggressive behaviors of the cancer cells in suspension, including cell aggregation, adhesion/attachment, and metastasis/invasion as well as anoikis resistance. Interaction between KIAA0100 and HSPA1A may be directly or indirectly responsible for imparting anoikis resistance to cancer cells, thus making KIAA0100 a potential target for chemotherapeutic intervention

## 4. Materials and Methods

### 4.1. Cell Cultures

MDA-MB-231 was maintained in L15 medium, supplemented with 10% FBS, 2 mM l-glutamine, 1 U/mL of penicillin, and 1 μg/mL of streptomycin at 37 °C and 0% CO_2_. HEK293 cells were maintained in DMEM with 2 mM l-glutamine, 1 U/mL of penicillin, and 1 μg/mL of streptomycin and 10% of FBS. pKIAA0100 (pKIA) expression vector with FLAG and Myc tag on its C-terminal was from Origene. HEK293 were transfected with pKIA by the Transfectamine 3000 reagent (ThermoFisher, Fremont, CA, USA) according to the manufacturer’s instruction. The stable cell line HEK293/pKIA was established by screening the transfected cells with 300 ug/mL Geneticin (ThermoFisher). For cell culture in suspension, the culture plates were coated with poly-HEMA as described before [71].

### 4.2. siRNA Transfection, Demecolcine Treatment

KIAA0100 siRNA (ThermoFisher) targeted to exon 25 (siRNA id: 219429) and siRNA targeted to exon 17 (siRNA id: 219430) were tested on MDA-MB-231 cells in the initial viability observation. After a similar result was obtained with each, only siRNA (219429) was used in the subsequent experiments. HSPA1A siRNA (siRNA id: 145248) targeted to exon 1, as well as the negative siRNA control (AM4635) that target no gene produce in human sequences were from ThermoFisher. Transfection of the siRNA was performed by the RNAiMAX transfection reagent from ThermoFisher according to the manufacturer’s instruction. Transfections were performed in either forward or reverse style. For forward transfection, cells were seeded on the culture plate and incubated overnight to let cells settle down and attached to the culture plate surface before transfection; for reverse transfection, transfection mixture was prepared and mixed with cells during seeding to the culture plate coated with poly-HEMA.

Demecolcine (Sigma, St. Louis, MO, USA) was dissolved in DMSO. HEK293 and HEK293/pKIA cells were seeded onto the 96 well tissue culture plate with 1, 10, 100, 1000 ng/mL (2.7, 27, 270, 2700 nM) final concentration of Demecolcine. Control for 0 ng/mL concentration was equal amount of DMSO instead. Cell viability were performed as described below at 0, 24, 48 and 72 h as described below and normalized to the 0 ng/mL control at each time point as 100% viability. For MDA-MB-231 cells, treatments of Demecolcine were at 0, 0.5, 5 and 50 µM concentrations and viability were assessed at 24 and 48 h after treatment.

### 4.3. Cell Growth/Viability, Attachment/Anchorage and Invasion/Metastasis assay

Cell Growth/Viability were tested either by Vi-Cell instrument (Beckman Coulter, Indianapolis, IN, USA) or multiTox-Fluor cell viability assay (Promega, Sunnyvale, CA, USA) according to the manufacturer’s instruction; Cell attachment/anchorage assays were performed by seeding the floating cell culture onto the culture plate, 12 h after the seeding, cells not able to attach/anchor to the plate surface were removed by gently washed with warm complete L15 medium, cell growth and viability were then tested as described above; Cell invasion/Metastasis was examined by the CultreCoat 96-Well BME-Coated Cell Invasion Optimization Assay (R&D systems, Minneapolis, MN, USA) according to the manufacturer’s instruction.

### 4.4. Real-Time Polymerase Chain Reaction (RT-PCR), Quantitative PCR (qPCR) and Western Blot and Immunoprecipitation (IP)

For RNA expression analysis, cells were lysed with TrisolLS reagent (ThermoFisher). RNAs were then purified by the RNA minElute kit from Qiagen. Reverse transcription was performed according to the Superscript III RT kit manual from ThermoFisher. For PCR amplification of full length KIAA0100, 4 sets of specific primers were used, set1: 5′-TCAAGGCTAGTGTGCAGGTG and 5′-GGTGCAGCCAGTGAGAGAAT, targeted 1029–1133 with product of 105 bp; set2: 5′-ACCAACCCAGCTCAGTATGC and 5′-CCTGACCCGTTGCTTCTTCT, targeted 5271–5337 with product of 105 bp; set3: 5′-TAATGGGTCGACTTGTGGGC and 5′-GGCCAATTAGTGTCCAGGCT, targeted 3103–3271 with a product of 207bp; set4: 5′-CGTTCCCGTCGTCTCTATGG and 5′-TCTAGCCCTGCTAAGCTCCA, targeted 2807–2877 with product of 71 bp. For RT-PCR of β-actin, 5′-ATATCGCCGCGCTCGTCGTC and 5′-TGGGCCTCGTCGCCCACATA primers with 166 bp product were used. For qPCR, Taqman probe assays for full length KIAA0100 and β-actin were from ThermoFisher performed according to the manufacturer’s recommendation; relative expression abundances was analyzed by the ΔΔCt method. For protein analysis, cells were also lysed with cell extraction buffer with protease inhibitor mixture (ThermoFisher), protein concentration was measured by microBCA assay (ThermoFisher). An equal amount of proteins were then separated on Tris-Acetate SDS-PAGE and transferred to the polyvinylidene fluoride (PDVF) membrane. Then a blot was detected by anti-KIAA0100 antibody (ThermoFisher), anti-Myc Tag antibody and anti-β-Actin antibody (Abcam, Cambridge, MA, USA).

HEK293/pKIA cells were washed by phosphate buffered saline (PBS), 6 mM of DTBP (dimethyl 3,3′-dithiobispropionimidate, ThermoFisher) in PBS were added to cover the cells and incubated for 5 min to crosslink the protein-protein interactions. Cells were then lysis in NP40 cell lysis buffer (ThermoFisher). Anti-FLAG monoclonal antibody (IgG1, Sigma) was used to capture the recombinant KIAA0100 protein; anti-HSPA1A monoclonal antibody (IgG1, Abcam) was used to capture the HSPA1A protein; both anti-Digoxigenin (Dig) monoclonal antibodies (IgG1, Abcam) were used as an isotype antibody, and bare beads were used as negative control. Equal amounts of cell lysate were immune-precipitated by the respective antibody and beads by Dynabead protein-G immunoprecipitation kit (ThermoFisher) according to the manufacturer’s instruction. In the end of the immunoprecipitation, the beads were mix with LDS buffer with reduce reagent and heated at 70 °C for 5 min. Protein samples were then separated on a 4–12% PAGE gel and transfer to PDVF membrane. The blot was then probed by goat anti-Myc tag antibody (Abcam), rabbit anti-α-Tubulin antibody (Abcam), rabbit anti-HSPA1A (Abcam), rabbit anti-HSP90AB1 antibody (Abcam), mouse anti-ACTB antibody (Abcam) respectively.

### 4.5. Mass Spectrometry Analysis

Protein samples were separated on 4–12% PAGE gel and stained with Brilliant Blue (ThermoFisher), slices containing the protein bands of interested were excised. In-gel digestions by trypsin (ThermoFisher) were then performed. Samples were analyzed by nanoflow reverse phase liquid chromatography using a Dionex Ultimate 3000 RSLCnano System (ThermoFisher) coupled in-line to a Q Exactive HF mass spectrometer (ThermoFisher). The nano LC (Liquid Chromatography) system included an Acclaim PepMap 100 C18 5 μm 100A 300 μm × 5 mm trap column and an EASY-Spray C18 2 μm 100 A 50 μm × 150 mm analytical column (ThermoFisher). Peptide samples were eluted with a two-step gradient of 2% to 30% B in 28 min then 30% to 45% B in 5 min, where B consisted of acetonitrile containing 0.1% formic acid. Blank samples consisting of 0.1% formic acid in water were injected between each sample and eluted with the same gradient profile and times as the samples. The LC system was interfaced with the mass spectrometry using an EASY-Spray electrospray ion source (ThermoFisher) and the samples were analyzed using positive ion spray voltage set to 2 kV, S-lens RF level at 65, and heated capillary at 285 °C. The Q Exactive HF was operated in the data-dependent acquisition mode for fragmentation. MS1 survey scans (*m*/*z* 400–1400) were acquired in the Orbitrap analyzer with a resolution of 120,000 at *m*/*z* 200, an accumulation target of 3 × 10^6^, and maximum fill time of 50 ms. MS2 scans were collected using a resolution of 30,000 at *m*/*z* 200, an accumulation target of 1 × 10^5^, and maximum fill time of 100 ms, with an isolation window of 1.5 *m*/*z*, normalized collision energy of 28, and charged state recognition between 2 and 7.

ProteoWizard was used for peak-picking, filtering out peaks with intensity less than 100 and converting the file to mzML format. Protein searches and identifications were performed using MS-GF+ search engine on Homo sapiens (Uniprot TaxID = 9606). Protein identification was set with parameter thresholds of peptide E-val of 0.01 and protein FDR of 0.01.

### 4.6. Annexin V Staining, Caspase 8, 3/7 Activity Assay

Anoikis was detected by Annexin V-FITC Apoptosis Detection Kit (Abcam) according to the manufacturer’s instruction. The images were taken by a Zeiss Axio Imager 2 Fluorescence Microscope. For caspase activity measurement, cells were lysis by the caspase buffer (Promega), protein concentration were then measured by microBCA assay. Equal amounts of protein (5 ug) were used for measuring caspase 8, 3/7 activities according to the instructions.

### 4.7. Immuno-Fluorescence

Cells were seeded on 8-well cell culture slides and incubated for designated time points and fixed with 4% formaldehyde for 10 min. The slides were then blocked by PBS buffer contains 5% of BSA. Anti-KIAA0100 C-terminal rabbit antibody (ThermoFisher) was co-labeled with anti α-tubulin monoclonal antibody (ThermoFisher) or anti-HSPA1A monoclonal antibody (Abcam) respectively, following by anti-rabbit-Alexa488 and anti-mouse-Alexa555 secondary antibodies, or Phalloidin-568 (ThermoFisher) for F-actin detection respectively. Slides were mounted by Prolong Gold anti-fade mountant with DAPI (4′,6-Diamidino-2-Phenylindole, Dihydrochloride, ThermoFisher). Images were acquired by a Zeiss Axio Imager 2 Fluorescence Microscope as well as Leica TCS SP5 AOBS Spectral Confocal System.

### 4.8. Statistical Analysis

All the experiments were performed at least three times. Mean, SD, and *t* test were calculated either using Excel software (*, *p* < 0.05). Graphs were generated in Excel.

## 5. Conclusions

Our study showed that silencing KIAA0100 expression was capable of reducing breast cancer cells’ aggressive behaviors, such as cell aggregation, re-attachment, invasion and most importantly, sensitizing cells to anoikis. We found that KIAA0100 regulates cancer cells’ resistance to anoikis by its close association with the stress protein HSPA1A.

## Supplementary Materials

The following are available online at http://www.mdpi.com/2072-6694/10/6/180/s1, Figure S1: Expression of full length KIAA0100 in three breast cancer cell lines. (**A**) RT-PCR of full length KIAA0100 by targeting different regions of the mRNA on MCF7, MDA-MB-231. 4 sets of primers targeted to different region of the full length KIAA0100 mRNA was used. All three cell lines were confirmed to express full length KIAA0100; (**B**) semi-quantitative mass spectrometry analysis of KIAA0100 in the three different cell lines, normalized globally. Expression of KIAA0100 in MCF7 > MDA-MB-231 > T47D, however, they are at similar range within 2 fold difference; (**C**) semi-quantitative mass spectrometry analysis of KIAA0100 in the three different cell lines, normalized by β-actin. Expression of KIAA0100 in MCF7 and MDA-MB-231 were similar while T47D was slightly lower, but they are all at similar level range with minor difference, Figure S2: Silencing expression of KIAA0100 in breast cancer cell T47D. T47D cells were transfected with KIAA0100 siRNA in a forward-transfection manner, qPCR was used to examine the reduction of KIAA0100 mRNA 24 hours after the treatment. Cell proliferation/growth was examined. (**A**) Real-time quantitative polymerase chain reaction (RT-qPCR) showed the efficiency of silencing the expression of KIAA0100 in T47D. 24 h after the treatment, mRNA level of KIAA0100 was reduced >80%.; (**B**) anchorag-dependent cell proliferation/growth was examined for up to 5 days after treatment, no significant difference between the Mock, neg siRNA control treated cell and the KIAA0100 siRNA treated cells, Figure S3: Silencing expression of KIAA0100 in breast cancer cell MDA-MB-231 by siRNA targeted to exon 17. (**A**) Total cell number in 24 and 48 h after treatment; (**B**) cell viability in 24 and 48 h after the treatment; (**C**) KIAA0100 RNA expression level in 24 and 48 h after the treatment by qPCR; (**D**) cell aggregation in 24 and 48 h after the treatment under microscope (*, *p* < 0.05), Figure S4: Immunofluorescence of native KIAA0100 protein in breast cancer cell T47D. KIAA0100 was co-immunostained with anti-KIAA010 antibody and Phalloidin-564 as well as DAPI. Native KIAA0100 stained as in the cytoplasm as speckles, the pattern is similar to that from MDA-MB-231, Figure S5: Immunofluorescence of native KIAA0100 protein in HEK293. KIAA0100 was immunostained with anti-KIAA010 antibody and DAPI. Native KIAA0100 stained as in the cytoplasm as speckles, the pattern of KIAA0100 is also similar to that from MDA-MB-231 as well as T47D.
