# Supplementary material for: KIAA0100 Modulates Cancer Cell Aggression Behavior of MDA-MB-231 through Microtubule and Heat Shock Proteins
Source: Cancers (Basel). 2018 Jun 4;10(6):180. doi: 10.3390/cancers10060180 (PMC6025110; doi:10.3390/cancers10060180)
Supplement: Supplementary file 1 [file cancers-10-00180-s001.pdf]

Supplementary Materials: KIAA0100 Modulates Cancer Cell Aggression Behavior of MDA-MB-231 through Microtubule and Heat Shock Proteins

Zhenyu Zhong, Vaishali Pannu, Matthew Rosenow, Adam Stark and David Spetzler

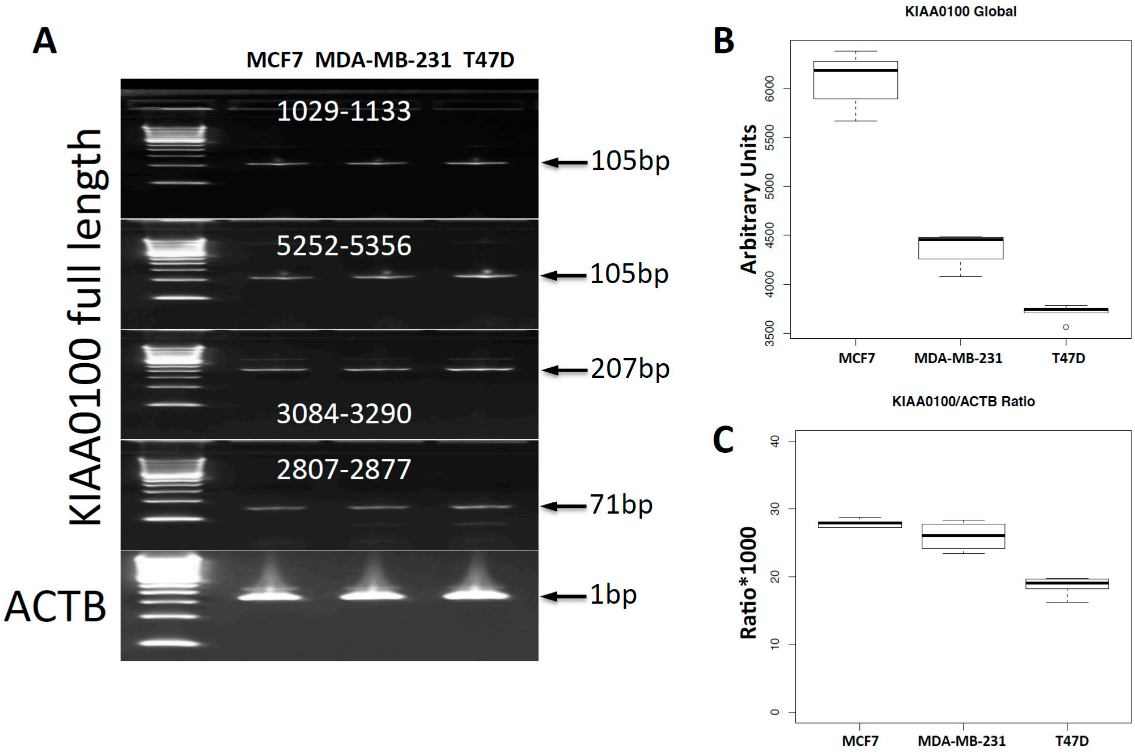

Figure S1. Expression of full length KIAA0100 in three breast cancer cell lines. (**A**) RT-PCR of full length KIAA0100 by targeting different regions of the mRNA on MCF7, MDA-MB-231. 4 sets of primers targeted to different region of the full length KIAA0100 mRNA was used. All three cell lines were confirmed to express full length KIAA0100; (**B**) semi-quantitative mass spectrometry analysis of KIAA0100 in the three different cell lines, normalized globally. Expression of KIAA0100 in MCF7> MDA-MB-231 > T47D, however, they are at similar range within 2 fold difference; (**C**) semi-quantitative mass spectrometry analysis of KIAA0100 in the three different cell lines, normalized by β-actin. Expression of KIAA0100 in MCF7 and MDA-MB-231 were similar while T47D was slightly lower, but they are all at similar level range with minor difference.

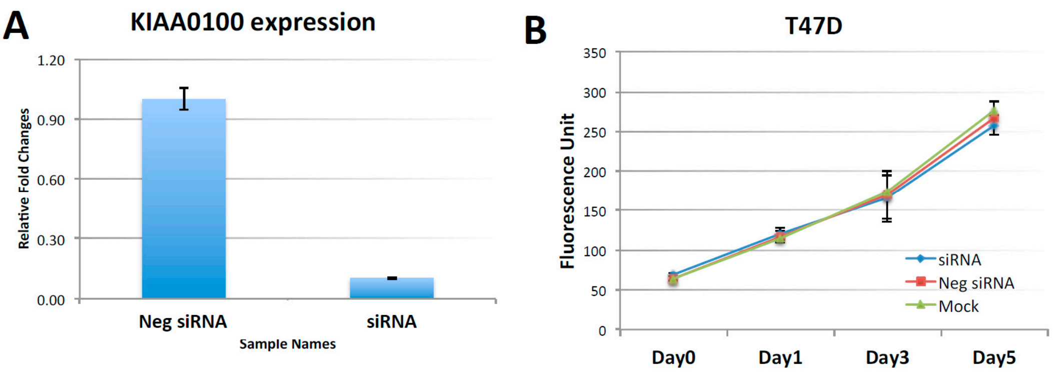

Figure S2 Silencing expression of KIAA0100 in breast cancer cell T47D. T47D cells were transfected with KIAA0100 siRNA in a forward transfection manner, qPCR was used to examine the reduction of KIAA0100 mRNA 24 hours after the treatment. Cell proliferation/growth was examined. (**A**) RT-qPCR showed the efficiency of silencing the expression of KIAA0100 in T47D. 24 hours after the treatment, mRNA level of KIAA0100 was reduced >80%.; (**B**) Anchorag-dependent cell proliferation/growth was examined for up to 5 days after treatment, no significant difference between the Mock, neg siRNA control treated cell and the KIAA0100 siRNA treated cells.

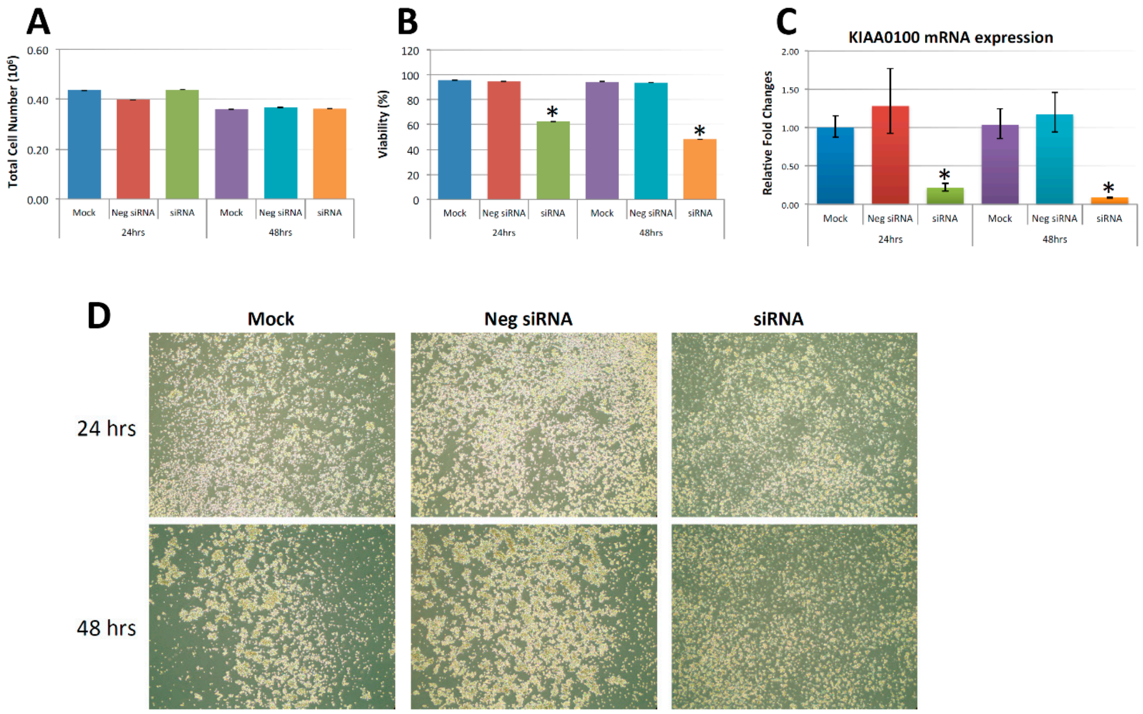

Figure S3. Silencing expression of KIAA0100 in breast cancer cell MDA-MB-231 by siRNA targeted to exon 17. (**A**) Total cell number in 24 and 48 hours after treatment; (**B**) Cell viability in 24 and 48 hours after the treatment; (**C**) KIAA0100 RNA expression level in 24 and 48 hours after the treatment by qPCR; (**D**) Cell aggregation in 24 and 48 hours after the treatment under microscope (* $p < 0.05$). Images were acquired at 40x.

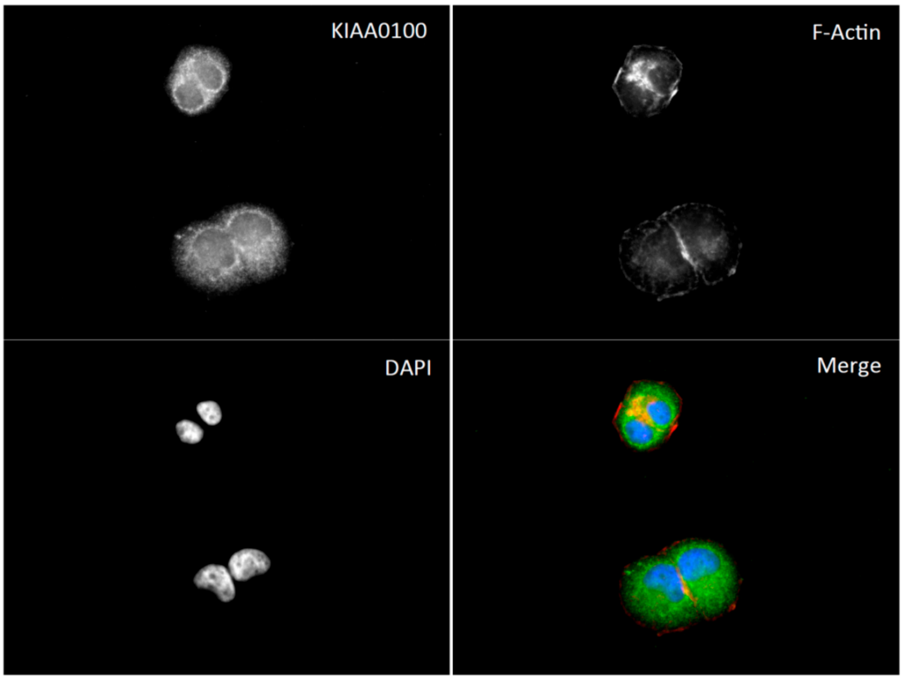

Figure S4. Immunofluorescence of native KIAA0100 protein in breast cancer cell T47D. KIAA0100 was co-immunostained with anti-KIAA010 antibody and Phalloidin-564 as well as DAPI. Native KIAA0100 stained as in the cytoplasm as speckles, the pattern is similar to that from MDA-MB-231. Images were acquired at 400x.

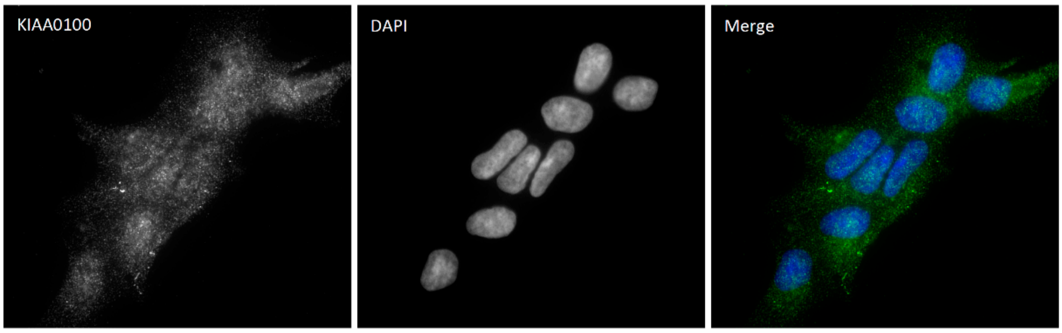

Figure S5. Immunofluorescence of native KIAA0100 protein in HEK293. KIAA0100 was immunostained with anti-KIAA010 antibody and DAPI. Native KIAA0100 stained as in the cytoplasm as speckles, the pattern of KIAA0100 is also similar to that from MDA-MB-231 as well as T47D. Images were acquired at 400x.

© 2018 by the authors. Licensee MDPI, Basel, Switzerland. This article is an open access article distributed under the terms and conditions of the Creative Commons Attribution (CC BY) license (http://creativecommons.org/licenses/by/4.0/).
